# Hybrid Surface Plasmon Polariton Wave Generation and Modulation by Chiral-Graphene-Metal (CGM) Structure

**DOI:** 10.1038/s41598-018-36241-2

**Published:** 2018-12-21

**Authors:** M. Z. Yaqoob, A. Ghaffar, Majeed Alkanhal, Sajjad ur Rehman, Faroq Razzaz

**Affiliations:** 1 Department of Physics, University of Agriculture, Faisalabad, Pakistan; 2 Department of Physics, Government College University, Faisalabad, Pakistan; 3 Department of Electrical Engineering, King Saud University, Riyadh, Saudi Arabia

## Abstract

Theoretical investigations are carried out to study hybrid SPP wave propagation along the Chiral-Graphene-Metal (CGM) interface. The Kubo formulism is used for the physical modeling of single-layer graphene and the impedance boundary conditions approach is applied at the CGM interface to compute the dispersion relationship for hybrid SPP waves. It is demonstrated that the chirality (*ξ*) and chemical potential (*μ*_*c*_) parameters can be used to modulate the resonance surface plasmon frequencies of the upper and lower propagating modes. Furthermore, the propagation bandgap between the upper and the lower modes can be tuned by changing the chirality parameter. The effect of the chemical potential (*μ*_*c*_)and the relaxation time (*τ*) on the normalized propagation constant, propagation length, and the effective refractive index is studied. The present work may have potential applications in optical and chiral sensing in the terahertz frequency range.

## Introduction

Atom-thick purely 2D carbon’s allotrope with a honeycomb structure, known as Graphene, is of interest in many research communities due to its extraordinary electronic, optical, chemical, and mechanical properties^1,2^. Its Dirac cone-like band structure with zero band gap distinguishes it from other ordinary materials because the electrons in graphene behave as massless fermions^3^. Recent developments in graphene-based optical devices have revolutionized optical societies by its ultrafast response^4^, broadband absorption^5^, and broadband communication^6^, terahertz lenses^7,8^, chemical and biological sensors^9^, ultra-broad photo detectors^10^, and terahertz phase shifters and modulators^11,12^.

Graphene optics exploits a new area of research, i.e. terahertz sciences, which deals with the propagation and modulation of terahertz waves for optical sensing, biochemical sensing, THz communication, and THz spectroscopy applications^13,14^. Wave propagation on the surface of graphene is evident from many theoretical and experimental studies that are commonly known as graphene plasmonics. The strongly confined modes and low loss propagation are remarkable features of graphene SPP waves compared to ordinary metal dielectric SPP waves^15^. Many researchers have studied different graphene-based structures for the exploitation of different types of surface wave and wave guide modes under different frequency ranges (i.e., planar structures^16^, graphene ribbons^17,18^, graphene coaxial cylindrical waveguides, and graphene shutters^19,20^).

Generally the organic, inorganic and biochemical molecules exhibit the phenomenon of dichroism which is scaled by the strength of chirality parameter, which, typically ranges from 10^−2^ to 10^−7^, depending upon their nature and structure^21,22^. Such molecules are modeled as chiral materials and have been the interest of many scientific communities as chiral sensing, chemical or analyte sensing tool and Enantiomer detection^23^.The chiral sculptured thin film-based metal dielectric interface geometry is examined for the optical sensing of an analyte by generating multiple SPP waves and Tamm Waves under different conditions^24–27^. Recently, the isotropic chiral-metal interface has been studied for the propagation of hybrid SPP wave generation for the detection of chiral sensing and enantiomeric detection it is reported that chirality is sensitive to the propagation length^28^. To further enhance the sensitivity, control and modulation of surface waves, the graphene’s dynamical tuning of conductivity, introduces new degrees of freedom to manipulate and control the optical properties of the graphene layer^15^. Due to these extraordinary properties, a graphene-based planar structure is motivated in the present work, i.e. the Chiral-Graphene-Metal (CGM) structure.

This paper investigates wave propagation along the planar CGM interface. Section 2 contains details of the mathematical formulation of the hybrid SPP wave propagation along the interface of the MGC structure, while Section 3 presents the results of numerical simulations to study the effect of chirality (*ξ*) and chemical potential (*μ*_*c*_) of single-layer graphene on the dispersion curve. The behavior of the normalized propagation constant, effective refractive index, and propagation length as a function of the frequency range are studied under different values of chirality (*ξ*), chemical potential (*μ*_*c*_) and relaxation time (*τ*). The time dependence is taken as *e*^*jωt*^ in all formulations.

## Formulations and Methodology

**Figure 1:**
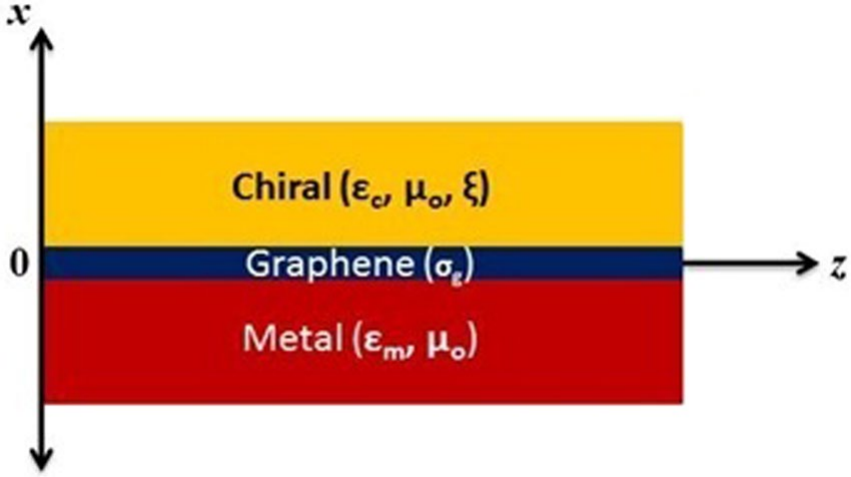
Chiral-Graphene-Metal geometry for hybrid Surface Waves.

The planar distribution of different materials and the mathematical formulation for the hybrid surface wave propagation along the CGM interface is given in this section. On the basis of the electromagnetic properties, the whole geometry can be divided in terms of three regions with respect to the material as region I represents the chiral material half space (*x* > 0) with parameters of the permittivity of chiral material (*ε*_*c*_), permeability (*μ*_*o*_), and chirality (*ξ*). While regions II and III represent the single-layer graphene (SLG) deposited on the metal half space *x* ≤ 0 with parameters *σ*_*g*_ as the graphene conductivity, *μ*_*o*_ as the permeability of free space and *ε*_*m*_ as the permittivity of metal to characterize the type of metal. In this geometry shown in Fig. 1. Due to the graphene’s purely two-dimensional geometry, it is assumed to have negligible thickness. The surface wave propagation is supposed to be along the z-axis and decay along the x-axis as it moves away from the interface.

The chiral material exhibits optical rotation phenomena and supports right circular polarization (RCP) and Left circular polarization (LCP) wave propagation, which means that the Transverse Magnetic (TM) and Transverse Electric (TE) modes cannot be studied independently, which is clear from the constitutive relationships i.e., ***D*** = *ε****E*** − *jξ****B*** and ***H*** = −*jξ****E*** + ***B***/*μ*^24,25^. The following phasors that qualify all the characteristics of chiral material are given below; the E_z_ and H_z_ fields components in chiral components can be express as the superposition of the two given modes^28^

1 $${{\rm{E}}}_{{\rm{z}}}=[{\psi }_{1}(x)+{\psi }_{2}(x)]{e}^{-{\rm{j}}{\rm{\beta }}z}$$

2 $${{\rm{H}}}_{{\rm{z}}}=j{\eta }_{c}[{\psi }_{1}(x)-{\psi }_{2}(x)]{e}^{-{\rm{j}}{\rm{\beta }}z}$$

where ${\psi }_{1}(x)={A}_{1}{e}^{-{k}_{1}x}$ and ${\psi }_{2}(x)={A}_{2}{e}^{-{k}_{2}x}$ are solutions to the wave equation that satisfy the wave equation when ${{\rm{k}}}_{1,2}=\sqrt{{\beta }^{2}-{{\rm{k}}}_{\pm }^{2}},$

$${\nabla }_{{\rm{x}}}^{2}{\psi }_{1,2}+\,({k}_{\pm }^{2}-{\beta }^{2}){\psi }_{1,2}=0$$

The remaining field components in the chiral medium can be obtained from coupled wave equation as

3 $${{\rm{E}}}_{{\rm{x}}}=-[({\rm{j}}{\rm{\beta }}/{k}_{1}){\psi }_{1}+({\rm{j}}{\rm{\beta }}/{k}_{2}){\psi }_{2}]{{\rm{e}}}^{-{\rm{j}}{\rm{\beta }}z}$$

4 $${{\rm{H}}}_{{\rm{x}}}={\eta }_{c}\sqrt{1+{\chi }^{2}}[({\rm{\beta }}/{k}_{1}){\psi }_{1}-({\rm{\beta }}/{k}_{2}){\psi }_{2}]{{\rm{e}}}^{-{\rm{j}}{\rm{\beta }}z}$$

5 $${{\rm{E}}}_{{\rm{y}}}=-[({k}_{+}/{k}_{1}){\psi }_{1}-({k}_{-}/{k}_{2}){\psi }_{2}]{{\rm{e}}}^{-{\rm{j}}{\rm{\beta }}z}$$

6 $${{\rm{H}}}_{{\rm{y}}}=-{\eta }_{c}\sqrt{1+{\chi }^{2}}[({k}_{+}/{k}_{1}){\psi }_{1}+({k}_{-}/{k}_{2}){\psi }_{2}]{{\rm{e}}}^{-{\rm{j}}{\rm{\beta }}z}$$

In above equations, *χ* = *ξ*/*η*_*c*_ is the normalized chirality admittance, *k*_±_ stands for the RCP and LCP propagation constants and expressed as ${k}_{\pm }={n}_{c}(\,\pm \,\chi \,+\,\sqrt{1+{\chi }^{2}}){k}_{o}$, where ${k}_{o}=\omega \sqrt{{\mu }_{o}{\varepsilon }_{o}}$. For the lower half space *x* < 0; i.e. the electromagnetic fields in metal are given below as

7 $$\begin{array}{c}{{\rm{E}}}_{{\rm{z}}}={{\rm{B}}}_{1}{{\rm{e}}}^{{\rm{\gamma }}x}{{\rm{e}}}^{-{\rm{j}}{\rm{\beta }}z}\\ {{\rm{H}}}_{{\rm{z}}}={{\rm{B}}}_{2}{{\rm{e}}}^{{\rm{\gamma }}x}{{\rm{e}}}^{-{\rm{j}}{\rm{\beta }}z}\end{array}\}$$

While the other associated field components can be derived by using Max well’s equation as reported by Mi & Van^28^

8 $${{\rm{E}}}_{x}=(j\beta /{\rm{\gamma }}){{\rm{E}}}_{{\rm{z}}}$$

9 $${{\rm{H}}}_{{\rm{x}}}=(j\beta /{\rm{\gamma }}){{\rm{H}}}_{{\rm{z}}}$$

10 $${{\rm{E}}}_{{\rm{y}}}=(-j\omega {\mu }_{0}/{\rm{\gamma }}){{\rm{H}}}_{{\rm{z}}}$$

11 $${{\rm{H}}}_{{\rm{y}}}=(-j\omega {\varepsilon }_{m}/{\rm{\gamma }}){{\rm{E}}}_{{\rm{z}}}$$

where ω is the incident frequency, *ε*_*m*_ and *μ*_0_ are the electric permittivity of metal and permeability of the free-space, $\gamma =\sqrt{{\beta }^{2}-{{\rm{k}}}_{{\rm{m}}}^{2}}$ is the decaying constant, and ${k}_{m}=\omega \sqrt{{\mu }_{0}{\varepsilon }_{m}}$, and A_1_, A_2_, B_1_ and B_2_ are unknown constants. To compute the dispersion relationship of the CGM interface, the impedance boundary condition approach is used to physically model the graphene-based interface as in Zhou *et al*.^17^. The boundary conditions with continuity at *x* = 0 are given as

12 $$\begin{array}{c}{e}_{x}\times ({{\boldsymbol{H}}}_{c}-{{\boldsymbol{H}}}_{m})={{\boldsymbol{J}}}^{s}={\sigma }_{g}{\boldsymbol{E}}\\ {e}_{x}\times ({{\boldsymbol{E}}}_{c}-{{\boldsymbol{E}}}_{m})=0\end{array}\}$$

where σ_g_ is the optical conductivity of graphene which is the function of incident frequency, chemical potential, scattering rates and temperature, as described by the Kubo formula^15^. Its mathematical modeling consists of two parts, inter-band & intra-band, which are given as:

13 $${{\rm{\sigma }}}_{{\rm{g}}}(\omega ,{\mu }_{c},{\rm{\tau }},{\rm{T}})=j\frac{{e}^{2}{{\rm{K}}}_{{\rm{B}}}T}{\pi {\hslash }^{2}(\omega +\frac{\,j}{{\rm{\tau }}})}(\frac{{\mu }_{c}}{{{\rm{K}}}_{{\rm{B}}}T}+2\,\mathrm{Log}[{e}^{-\frac{{\mu }_{c}}{{{\rm{K}}}_{{\rm{B}}}T}}+1])+j\frac{{e}^{2}}{4{\rm{\pi }}\hslash }\,\mathrm{Log}[\frac{2|{\mu }_{c}|-\hslash (\omega +\frac{\,j}{{\rm{\tau }}})}{2|{\mu }_{c}|+\hslash (\omega +\frac{\,j}{{\rm{\tau }}})}]$$

where *ħ* is the reduced Planck constant, *e* is the electronic charge, *τ* is the momentum relaxation time, *μ* is the chemical potential, *ω*is the optical frequency, *k*_*B*_ is the Boltzmann constant, and *T* is the temperature. The chemical potential can be obtained as ${\mu }_{c}=h{v}_{F}\sqrt{\pi n}$ under terahertz range, where the *v*_*F*_ is the Fermi velocity and *n* is the carrier density. It is clear that chemical potential of graphene can be tuned by electronically gated voltage or by doping of carrier density from 0 eV to 2 eV and much higher values has been discussed in^29,30^. The relaxation time is associated with the quality of graphene and phenomenological scattering rate as reported by Idzuchi *et al*.^31^.

The following dispersion relationship for the CGM interface is obtained after implementing the above boundary conditions.

14 $$\begin{array}{c}({k}_{+}\gamma +{k}_{1}{\eta }_{c}\omega {\mu }_{o}\sqrt{1+{X}^{2}})({k}_{-}\gamma +\frac{{k}_{2}\omega {\varepsilon }_{m}}{\sqrt{1+{X}^{2}}{\eta }_{c}})+{k}_{1}{k}_{-}(\frac{j{\omega }^{2}{\varepsilon }_{o}{\mu }_{o}{\sigma }_{g}+\,j{\gamma }^{2}({\sigma }_{g}-{{\sigma }_{g}}^{2})}{\sqrt{1+{X}^{2}}{\eta }_{c}})\\ \,\,+2\,j{k}_{1}{k}_{2}{\sigma }_{g}=-\,({k}_{-}\gamma +{k}_{2}{\eta }_{c}\omega {\mu }_{o}\sqrt{1+{X}^{2}})({k}_{+}\gamma +\frac{{k}_{1}\omega {\varepsilon }_{m}}{\sqrt{1+{X}^{2}}{\eta }_{c}})\\ \,\,-{k}_{2}{k}_{+}(\frac{j{\omega }^{2}{\varepsilon }_{o}{\mu }_{o}{\sigma }_{g}+j{\gamma }^{2}({\sigma }_{g}-{{\sigma }_{g}}^{2})}{\sqrt{1+{X}^{2}}{\eta }_{c}})-2\,j{k}_{+}{k}_{-}{\sigma }_{g}\end{array}$$

If the graphene monolayer is removed i.e., *σ*_*g*_ = 0,then the above dispersion equation transforms into the dispersion relation presented in Mi and Van^28^. The next section presents simulation results for further studying the physical behavior of hybrid surface wave propagating modes.

## Results and Discussion

Numerical simulations are executed in the Wolfarm Mathematica® software package, to explore the characteristics of hybrid SPP wave propagation along the CGM interface. Generally the organic and biochemical materials has refractive index from 1 to 1.6 as given in^32,33^. Therefore, in present simulations these values will be used. In all graphs, gold is assumed as the supporting metal with its plasma frequency *ω*_*p*_ = 1.30 × 10^16^ rad/s and damping factor *γ* = 2.80 × 10^13^ rad/s as described by the Drude Model^28^.

**Figure 2:**
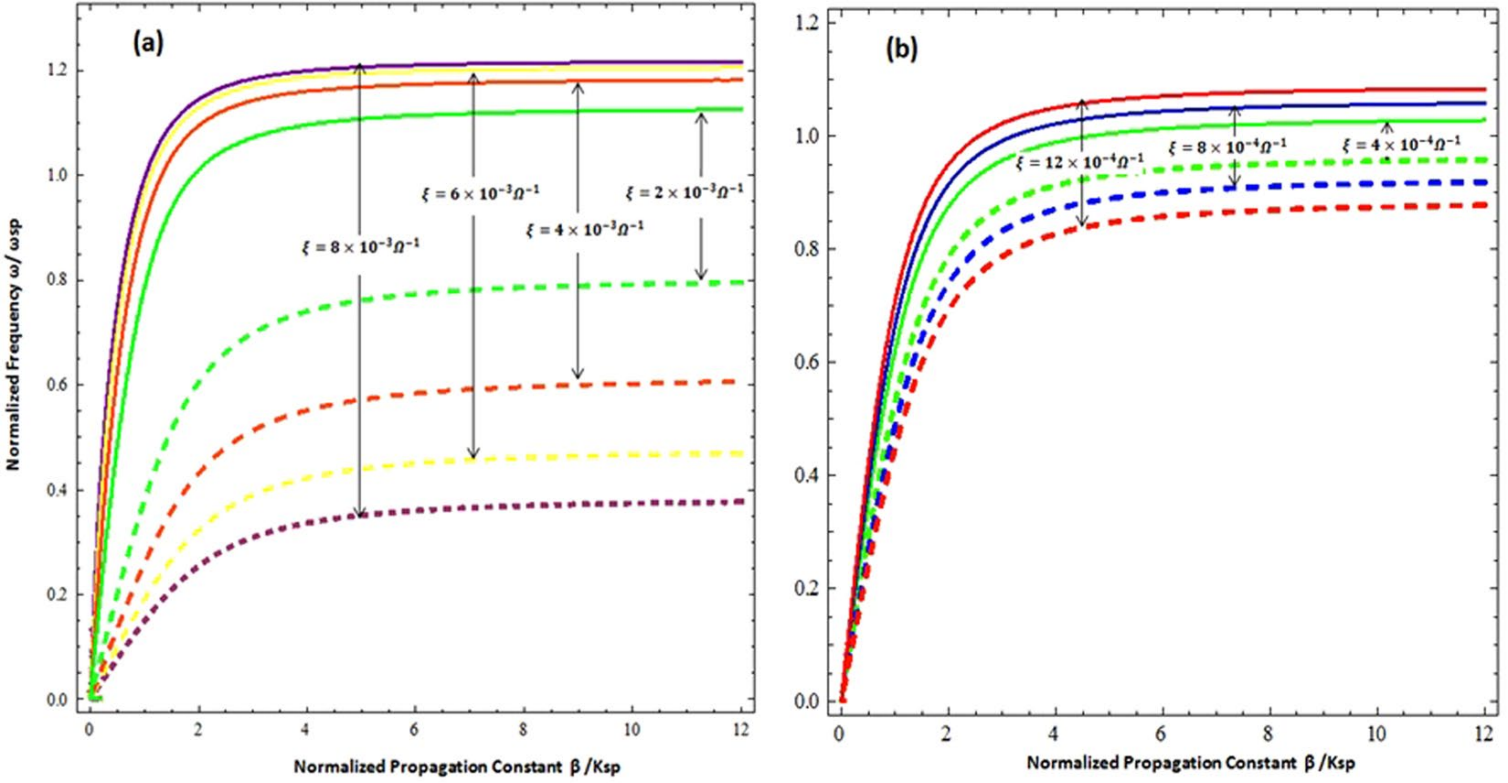
Dispersion relation of the Chiral-Graphene-Metal interface under different chirality parameter (*ξ*) (**a**) for higher values of *ξ* (**b**) for lower values of *ξ* with *μ*_*c*_ = 0.3 eV, *T* = 300 *K* and *τ* = 1.66 ps.

**Figure 3:**
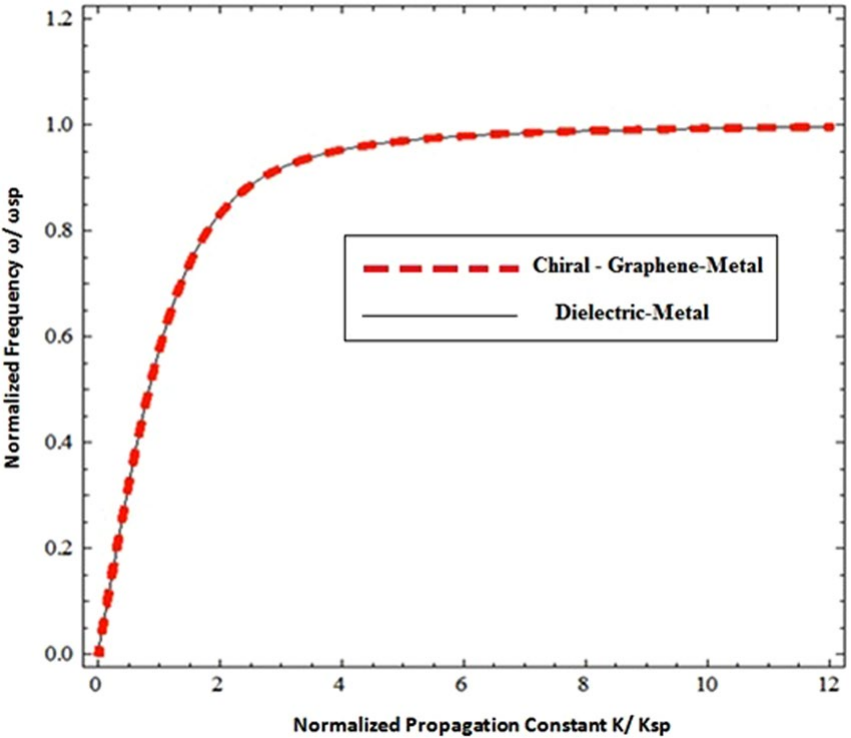
Dispersion relation of surface waves at Chiral-Graphene-Metal interface under special conditions (*ξ* = 0.0 *Ω*^−1^ & *σ*_*g*_ = 0).

**Figure 4:**
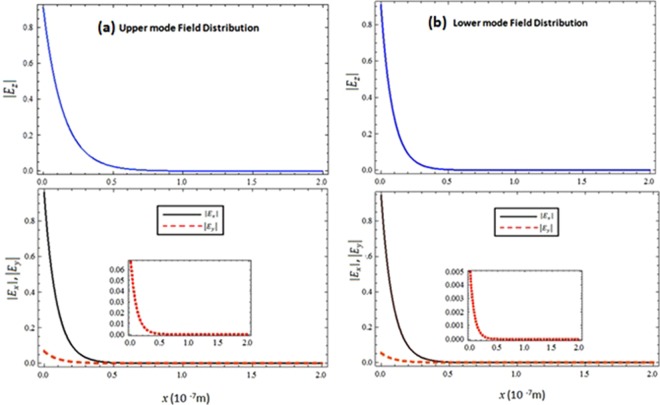
Field distribution of Hybrid SPP waves in chiral media (**a**) |*E*_*z*_| and (**b**) |*E*_*x*_|,|*E*_*y*_| with *ξ* = 1 × 10^−4^*Ω*^−1^, *μ*_*c*_ = 0.3 eV, *T* = 300 *K* and *τ* = 1.66 ps.

To study the possible modes of propagation along the CGM interface, the dispersion relationship is plotted between the normalized frequency (*ω*/*ω*_*sp*_) and the normalized propagation constant (*β*/*k*_*sp*_) in Fig. 2. The frequency (*ω*) is normalized by the surface plasmon resonance frequency ${\omega }_{sp}=\frac{{\omega }_{p}}{\sqrt{1+{{n}_{c}}^{2}}}$ and the propagation constant (*β*) is normalized by ${k}_{sp}=\frac{{\omega }_{sp}}{c}$. It is obvious from the Fig. 2(a,b) that the geometry supports two different propagation modes i.e. the upper mode with a high frequency and the lower with a low frequency, which are collectively known as hybrid modes. The propagation bandgap between the upper and lower mode is highly dependent on the value of chirality. Under high order chiral values (i.e.***ξ*** **=** **2** **×** **10**^**−3**^ ***Ω***^**−1**^, ***ξ*** **=** **4** **×** **10**^**−3**^ ***Ω***^**−**1^, ***ξ*** **=** **6** **×** **10**^**−3**^***Ω***^**−1**^, and ***ξ*** **=** **8** **×** **10**^**−3**^ ***Ω***^**−1**^) it can be observed that the propagation bandgap between modes increases in proportion to the increase of chirality and decreases with the decrease in strength of the chirality parameter. The propagation band gap between the upper and lower can be tuned by tuning the chirality of the chiral medium. Further, it is clear that with the decrease of chirality i.e., low order chiral values (***ξ*** **=** **4** **×** **10**^**−4**^ ***Ω***^**−1**^, ***ξ*** **=** **8** **×** **10**^**−4**^***Ω***^**−1**^ and ***ξ*** **=** **12** **×** **10**^**−4**^ ***Ω***^**−1**^) the forbidden region between the modes starts squeezing as presented in the Fig. 2(b) and they approach each other with the decrease of chirality. Under special conditions (i.e. for zero chirality when *ξ* = 0.0*Ω*^−1^ & *σ*_*g*_ = 0) the propagation band gap between the upper and lower modes becomes zero and the dispersion curve only presents a single mode of propagation (i.e. the standard dielectric Surface Plasmon Polariton (SPP)^28^ as depicted in Fig. 3. This confirms the accuracy of our numerical simulation results. Furthermore, it confirms that the chirality (electromagnetic coupling of chiral media) is responsible for the generation of hybrid modes and the propagation band gap between them. To further verify the physical existence of hybrid SPP waves, the field distribution of surface wave in chiral media of upper and lower modes has been presented in the Fig. 4(a,b) respectively. All the fields are normalized by the peak value of |*E*_*x*_|. The Fig. 4 confirms existence of hybrid SPP waves and also shows that hybrid surface waves start decaying exponentially as move away from the interface, which is the one of the fundamental characteristics of surface waves. It is also clear from the fig. that the upper mode and lower mode field distribution has quite similar behavior but the upper mode has higher amplitude of |*E*_*y*_| component as compared to lower mode.

**Figure 5:**
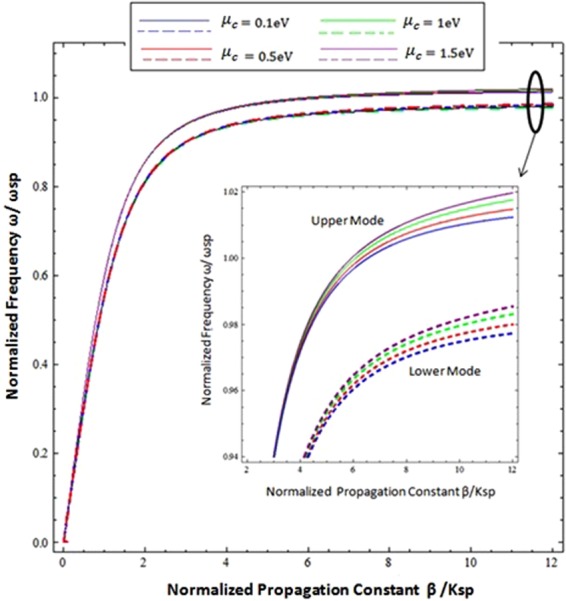
Influncence of chemical poetential on the dispersion curve of hybrid surface waves at Chiral-Graphene-Metal interface with *ξ* = 2 × 10^−4^ *Ω*^−1^, *T* = 300 *K* and τ = 1.66 ps.

Figure 5 presents the effect of the chemical potential on the upper and lower hybrid propagating modes along the CGM interface. The chemical potential *μ*_*c*_ of graphene can be tuned by gate voltage or doping^15^, the chemical potential has different values in these dispersion curves i.e. *μ*_*c*_ = 0.1eV, *μ*_*c*_ = 0.5eV, *μ*_*c*_ = 1.0 eV, and *μ*_*c*_ = 1.5 eV. Comparison confirms that the chemical potential also plays a significant role in the modulation of hybrid modes; the resonance frequency for the upper and lower modes increases with the increase of chemical potential.

**Figure 6:**
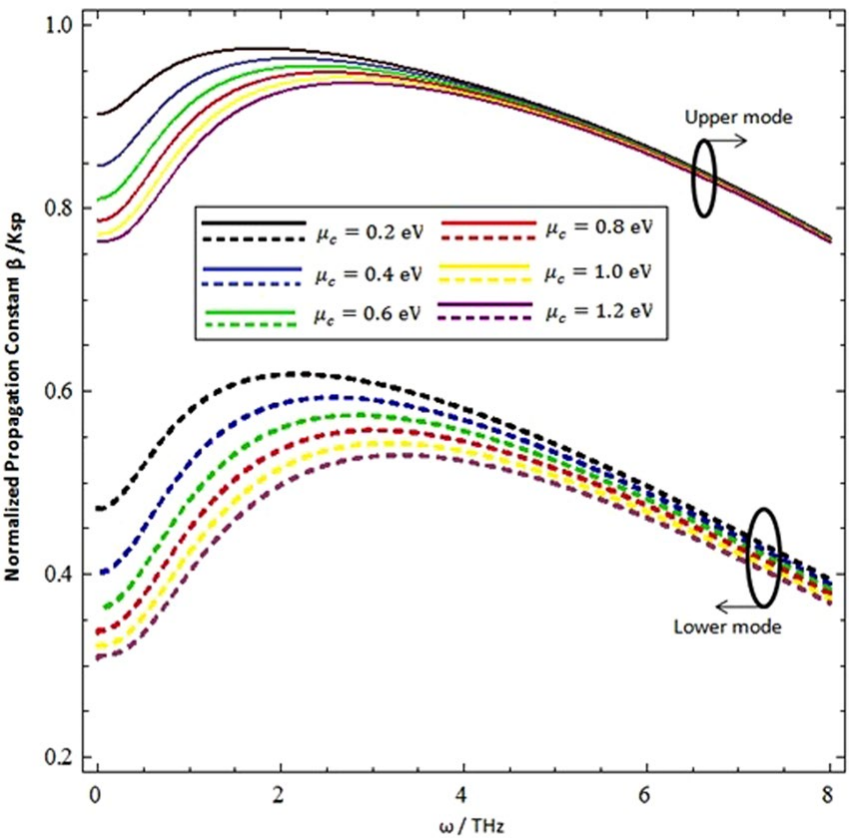
Influncence of chemical poetential on the normalized progationconstatnt as fucntion of incidence frequency at *ξ* = 3 × 10^−3^ *Ω*^−1^, = 300*K*, and *τ* = 1.66 ps.

The graphene mainly contributes in the terahertz region for the propagation of surface wave generation, thus the influence of graphene parameters (i.e. the chemical potential and relaxation time (*τ*) of the intraband) on the hybrid modes of surface wave propagation for the normalized wave propagation constant (*β*/*k*_*sp*_) is plotted under the terahertz frequency range (0.1–8 THz) and presented in Figs 5 and 6. Figure 5 exhibits the nonlinear behavior of both hybrid upper and lower modes in the terahertz frequency range for different chemical potential values (i.e. *μ*_*c*_ = 0.2 eV, *μ*_*c*_ = 0.4 eV, *μ*_*c*_ = 0.6 eV, *μ*_*c*_ = 0.8 eV, *μ*_*c*_ = 1.0 eV & *μ*_*c*_ = 1.2 eV). The influence of the chemical potential on upper modes is more dominant at low frequencies compared to high terahertz frequencies, while the lower modes have a significant difference in all terahertz frequencies.

**Figure 7:**
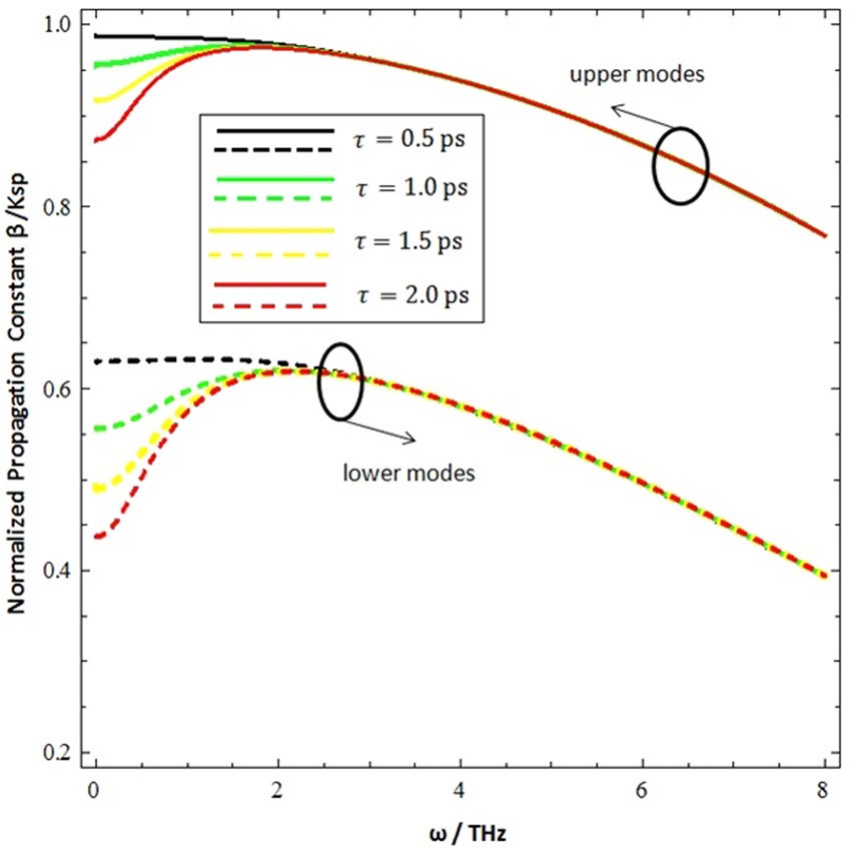
Influncence of relaxation time (*τ*) on the normalized progation constatnt as fucntion of incidence frequency at *ξ* = 3 × 10^−3^ *Ω*^−1^, *T* = 300 *K* and *μ*_*c*_ = 0.3 *eV*.

Figure 7 represents the influence of the relaxation time (i.e.*τ* = 0.5 ps,*τ* = 1.0 ps, *τ* = 1.5 ps, and *τ* = 2.0 ps) on the normalized propagation constant (*β*/*k*_*sp*_) under the terahertz frequency range (0.1–8 THz). The normalized propagation constant (*β*/*k*_*sp*_) for upper modes has a significant effect at 0.1–2 THz but the relaxation time has no effect on the wave propagation constantfor frequencies higher than 2 THz. While the lower modes have a distinct wave propagation constant in the 0.1–3 THz range above this frequency, the relaxation time has no effect on the wave propagation constant.

**Figure 8:**
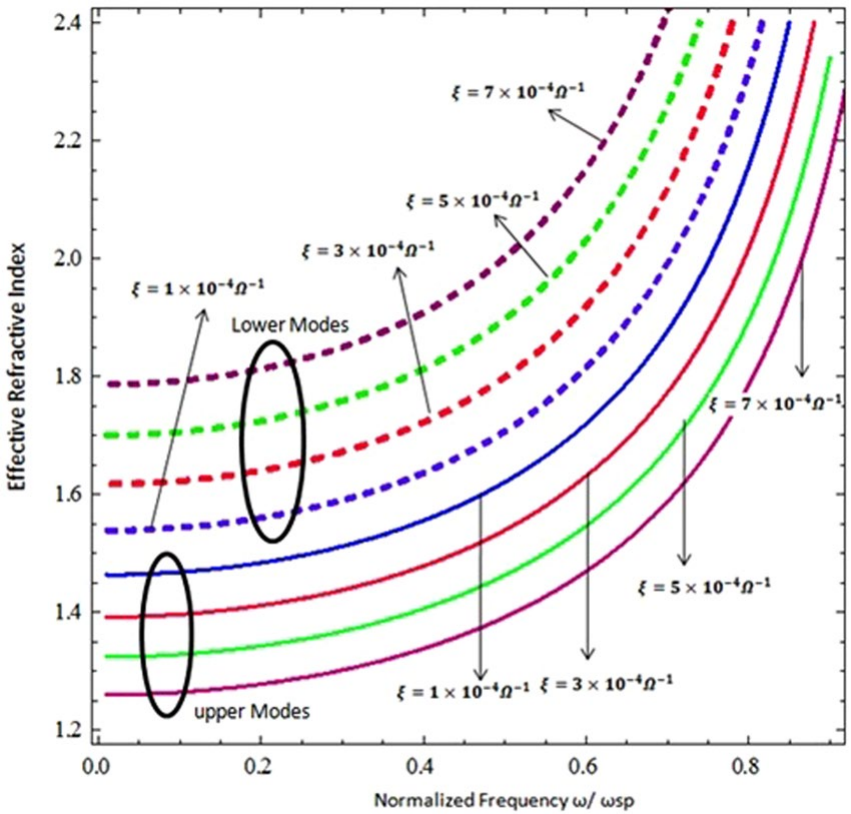
Effective Refractive index as function of normalized frequency with parameters i.e., *μ*_*c*_ = 0.6 eV, *T* = 300 *K*, and *τ* = 3.0 ps.

The effective refractive index (*N*_*eff*_ = *Re*{*β*}/*k*_*o*_) as a function of the normalized frequency (*ω*/*ω*_*sp*_) is presented in Fig. 8. The chirality and chemical potential of the CGM structure plays a crucial role in the modulation of the hybrid surface upper and lower modes by changing their effective refractive index. The effective index varies within 1.25–1.46 for the upper modes and 1.55–1.80 for the lower modes at different values of chirality (***ξ*** **=** **1** **×** **10**^**−4**^ ***Ω***^**−1**^, ***ξ*** **=** **3** **×** **10**^**−4**^ ***Ω***^**−1**^, ***ξ*** **=** **5** **×** **10**^**−4**^ ***Ω***^**−1**^ & *ξ* = 7 × 10^−4^ *Ω*^−1^) and chemical potential (*μ*_*c*_ = 0.6 eV). The change in the effective refractive index can be used to modulate the propagating phase speed of different modes at different values of chiral and graphene parameters along the CGM interface, which is clear in Fig. 8. With the increase of normalized frequency (*ω*/*ω*_*sp*_), the effective refractive index (*N*_*eff*_) increases and the phase speed of the propagating hybrid modes decreases.

**Figure 9:**
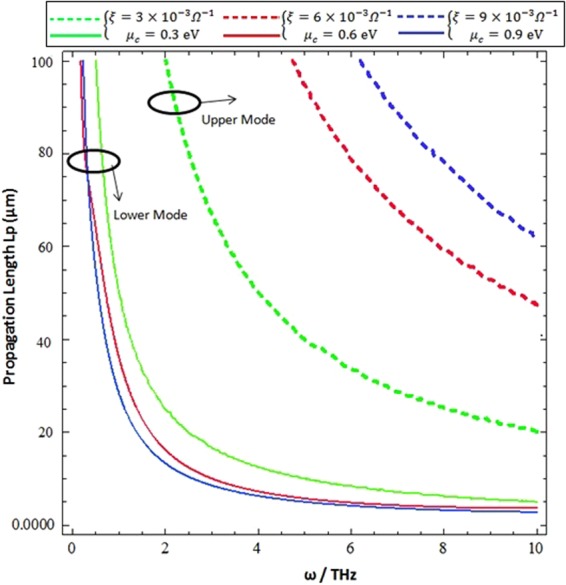
Influence of chemical potential and chirality on the propagation length of hybrid surface wave modes as function of incidence frequency.

The propagation length ${L}_{P}=\frac{1}{2Im\{\beta \}}$ as a function of the incidence terahertz frequency (*ω* = 0.1 − 10 THz) for different values of chirality (*ξ* = 3 × 10^−3^ *Ω*^−1^, 6 × 10^−3^ *Ω*^−1^ & 9 × 10^−3^ *Ω*^−1^) and chemical potential (*μ*_*c*_ = 0.3 eV, 0.6 eV, and 0.9 eV) is presented in Fig. 9. It is clear that chirality (*ξ*) and the chemical potential (*μ*_*c*_) can significantly modulate the propagation length of both hybrid modes. With the increase of chirality and chemical potential,the propagation length of the upper mode increases in contrast to the lower propagating modes. The propagation length profiles of the upper and lower modes follow the exponential decay. The incorporation of graphene can be used to increase the propagation length of hybrid modes compared to ordinary chiral-metal interface modes.

**Figure 10:**
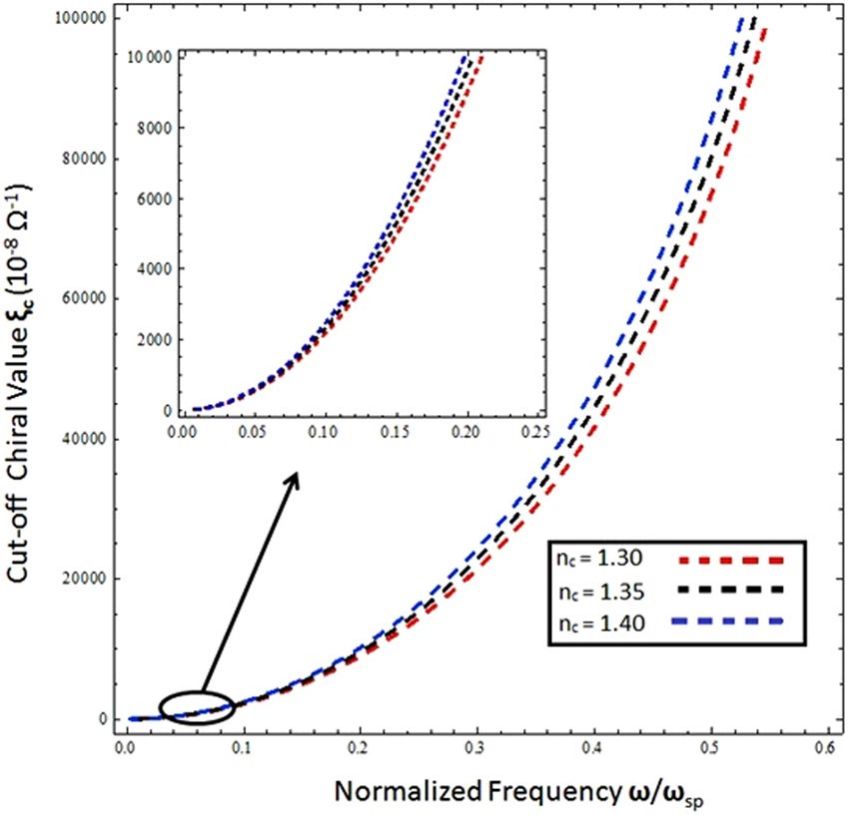
Cut-off Chiral values as function of normalized frequency under different values of refractive index with *μ*_*c*_ = 0.2 eV, *T* = 300 *K*, and *τ* = 1.6 ps.

The Fig. 10 shows the relation of cut-off chiral values (ξ_c_) as function of normalized frequency (*ω*/*ω*_*sp*_) under different values of refractive index i.e., (n_c_ = 1.3, 1.35 and 1.4). The cut off condition is obtained by assuming the *n*_+_ > *n*_−_ and setting the values of *β* = *k*_+_, *k*_*x*1_ = 0 and ${k}_{x2}={({{k}_{+}}^{2}-{{k}_{-}}^{2})}^{1/2}\,$in equation (14) as provided by^28^. The cut off chiral values varied from 10^−8^ to 10^−3^ Ω^−1^, and it is obvious from fig. that it exhibits the sensitive behavior towards the chemical and optical changes in the chiral material.With the increase of normalized frequency the cut off chiral values increases and for lower frequency range shows adistinct behavior for different values of refractive index.In conclusion of these results, proposed scheme can be used for analyzing the different analyte on the basis of their chiroptic and chemical properties.

## Conclusion

The CGM interface is used to excite the hybrid SPP waves. The dispersion relationship is computed by implementing the impedance boundary conditions approach andthe following conclusions can be drawn:

(i) Hybrid surface wave modes (the upper and lower) propagate along the CGM interface.
(ii) The frequency difference between the upper and lower modes (viz. the propagation bandgap) can be tuned by changing the chirality or chemical potential of graphene.
(iii) The normalized propagation constant is very sensitive to graphene’s chemical potential and time relaxation for the lower terahertz frequency range, but these parameters have no significant effect at the higher terahertz range.
(iv) The effective mode index and propagation length as a function of the terahertz frequency range is studied and concluded that under appropriate parameters one can modulate the effective refractive index, the phase speed of surface waves, and the propagation length. The present method of surface wave modulation is quite simple as compared to corrugated structures^34^.
(v) The proposed geometry can be used for optical sensing and wave propagation in the terahertz regime.
